# TENG-Based Self-Powered Silent Speech Recognition Interface: from Assistive Communication to Immersive AR/VR Interaction

**DOI:** 10.1007/s40820-025-01982-z

**Published:** 2026-01-12

**Authors:** Shuai Lin, Yanmin Guo, Xiangyao Zeng, Xiongtu Zhou, Yongai Zhang, Chengda Li, Chaoxing Wu

**Affiliations:** 1 School of Physics and Information Engineering, Fuzhou University, Fuzhou, 350108 People’s Republic of China; 2 Fujian Science & Technology Innovation Laboratory for Optoelectronic Information of China, Fuzhou, 350116 People’s Republic of China; 3 School of Artificial Intelligence, Xiamen City University, Xiamen, 361008 People’s Republic of China

**Keywords:** Flexible pressure sensor, Silent speech recognition, Triboelectric nanogenerator, Deep learning, AR/VR interaction

## Abstract

Lip language provides a silent, intuitive, and efficient mode of communication, offering a promising solution for individuals with speech impairments. Its articulation relies on complex movements of the jaw and the muscles surrounding it. However, the accurate and real-time acquisition and decoding of these movements into reliable silent speech signals remains a significant challenge. In this work, we propose a real-time silent speech recognition system, which integrates a triboelectric nanogenerator-based flexible pressure sensor (FPS) with a deep learning framework. The FPS employs a porous pyramid–structured silicone film as the negative triboelectric layer, enabling highly sensitive pressure detection in the low-force regime (1 V N^− 1^ for 0–10 N and 4.6 V N^− 1^ for 10–24 N). This allows it to precisely capture jaw movements during speech and convert them into electrical signals. To decode the signals, we proposed a convolutional neural network-long short-term memory (CNN–LSTM) hybrid network, combining CNN and LSTM model to extract both local spatial features and temporal dynamics. The model achieved 95.83% classification accuracy in 30 categories of daily words. Furthermore, the decoded silent speech signals can be directly translated into executable commands for contactless and precise control of the smartphone. The system can also be connected to AR glasses, offering a novel human–machine interaction approach with promising potential in AR/VR applications.

**Supplementary Information:**

The online version contains supplementary material available at 10.1007/s40820-025-01982-z.

## Highlights

- A porous pyramid-structured triboelectric nanogenerator sensor is designed for self-powered silent speech signal acquisition.
- A hybrid neural network that combines convolutional neural network with long short-term memory is proposed to accurately decode silent speech signals.
- Silent speech commands enable real-time, contactless control of smartphones and immersive AR/VR interaction.

**Supplementary Information:**

The online version contains supplementary material available at 10.1007/s40820-025-01982-z.

## Graphical abstract

**Figure:**
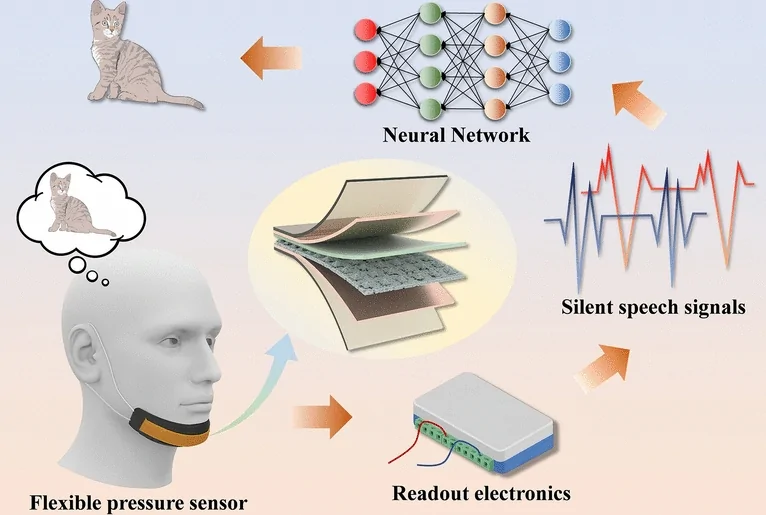

## Introduction

Language, the cornerstone of human connection, is essential for expressing thoughts and building social bonds [1–3]. Yet, for millions with speech impairments due to neurological disorders, brain injuries, or congenital conditions [4–14], the inability to vocalize severely limits social participation and access to services [15]. Silent speech technologies, particularly lip-based communication, offer a critical alternative for these individuals to reclaim their voice.

Lip language provides a natural, intuitive, and hands-free means of silent speech communication [16–19]. Importantly, its articulation involves not only the lips but also the jaw and the muscles surrounding it, whose kinematic patterns carry essential information for recognizing silent speech. Despite its potential, accurately capturing and decoding these subtle articulatory movements remains a significant technical challenge, especially in real-world conditions.

Currently, silent speech recognition (SSR) methods mainly include vision-based, electromyography (EMG)-based, and radar-based techniques, which have achieved significant breakthroughs in recent years. Vision-based methods have leveraged multimodal fusion and deep learning, such as Yu et al.’s cascade fusion algorithm with pre-trained Visual-HuBERT for integrating tongue and lip features [20], and Wang et al.’s PointVSR model using depth-sensed point cloud data from multiple sensor positions [21]. EMG-based methods have explored novel combinations of time–frequency features, deep learning architectures, and signal-to-image transformations, including Huang et al.’s GRU-based modeling on a Chinese word corpus [22] and Li et al.’s SVIT-SSR framework employing Vision Transformers [23]. Radar-based methods have demonstrated the potential of non-contact recognition. Menezes et al. explored continuous phoneme recognition with radar signals using feature combinations and CNN-MLP models [24], and further investigated on-body antenna configurations to optimize multi-speaker recognition [25]. These studies collectively highlight recent innovations in multimodal fusion, model design, and non-contact recognition, providing a foundation for advancing SSR technologies.

However, each method has its own application scenarios and challenges. Vision-based method provides rich articulatory information in well-lit, unobstructed environments, achieving high accuracy, but is susceptible to lighting variations and occlusion. EMG-based method achieves high sensitivity by detecting muscle activity directly, yet requires skin-attached electrodes and external power. Radar-based method enables non-contact detection and can operate under low-light or occluded conditions, although its spatial resolution is moderate and it is sensitive to electromagnetic interference (Table S1).

Since it was first proposed in 2012 [26, 27], the triboelectric nanogenerator (TENG) has rapidly become a technology of great interest due to its unique self-powered characteristics, low cost, and easy fabrication [28–31]. TENG converts mechanical energy into electrical energy through the principle of the triboelectric effect and electrostatic induction, showing potential application in human–machine interaction [32–36]. For example, by using this technology, accurate recognition of large-range joint motions can be achieved, enabling self-powered control of robotic devices through neck gestures [37]. Due to its high sensitivity, self-powered operation, and wearable compatibility, TENG shows strong potential for applications in real-time silent speech recognition.

Here, we report a real-time silent speech recognition system (RT-SSRS) that integrates a self-powered flexible pressure sensor (FPS) based on TENG with a deep learning framework. The FPS employs a porous pyramid-structured silicone (PPS) film as the negative triboelectric layer, designed to precisely capture jaw movements during speech. To decode these complex spatiotemporal signals, we proposed a hybrid neural network that combines convolutional neural network (CNN) for spatial feature extraction with long short-term memory (LSTM) for capturing temporal dynamics. This model achieves a classification accuracy of 95.83% across 30 daily words. Furthermore, we demonstrate the practical utility of RT-SSRS in real-world human–machine interaction scenarios, translating silent speech commands into contactless smartphone control actions. The system can also be connected to AR glasses, demonstrating a potential prototype for future human–machine interaction in AR/VR applications. Compared to other methods, our TENG-based approach demonstrates high sensitivity to subtle pressure variations, lightweight and comfortable wearable design, self-powered operation, and excellent environmental robustness (Table S1). Moreover, all components of our sensor are made from common, low-cost materials, providing biocompatibility, mechanical flexibility, and scalability. These characteristics make it highly suitable for integration into AR/VR interaction systems. With further optimization in the future, we believe there will be further breakthroughs in accuracy and other aspects. Overall, the RT-SSRS ensures reliable silent speech recognition for speech-impaired users, reduces communication burden, and offers a novel human–machine interaction approach with broad application prospects and significant societal value.

## Experimental Section

### Fabrication of the PPS Film

The PPS film was prepared using a simple and efficient sacrificial NaCl template method. First, component A and component B of Ecoflex 00–30 were mixed in a 1:1 weight ratio. Subsequently, NaCl particles were added to the prepared Ecoflex mixture in a 1:1 weight ratio with continuous stirring for 20 min to allow for thorough mixing. Next, the stirred Ecoflex-NaCl mixture was poured into a mold with a pyramidal structure and vacuumed to completely remove air bubbles. The mixture was then cured on a heating table at 60 °C for 1 h to ensure that the mixture was fully cured and a stable pyramid structure was formed. Once curing was complete, the Ecoflex-NaCl mixture was carefully peeled from the mold using tweezers and immersed in deionized water for 12 h, during which time the water was changed periodically to accelerate the dissolution of the NaCl particles, resulting in the formation of a porous structure. Finally, the soaked samples were thoroughly rinsed with deionized water to remove residual salts and other impurities. After cleaning, the samples were dried in an oven at 100 °C for 12 h to ensure that their internal water was completely evaporated, and finally PPS film were obtained.

### Fabrication of the FPS

The PPS film and nylon were used as the negative and positive triboelectric layers, respectively, and were cut into rectangular pieces with dimensions of 2 cm × 8 cm. Then, copper foils of the same size were pasted as electrodes, and a conductive copper wire was led out from each of them to facilitate the connection with external circuits. Next, the PPS film was placed opposite to the nylon, and the entire sensor was encapsulated with a polyimide (PI) film to mitigate the effects of the external environment during use while maintaining its flexibility and durability. Finally, a 3 cm × 16 cm piece of fabric was cut, rubber bands were tied at both ends, and the FPS was fixed to the fabric.

### Characterization and Measurements

A linear motor (LinMot B 01–37 × 166/260) was used to generate periodic reciprocating motion for applying pressure. The pressure magnitude was adjusted by varying the movement distance and measured using a force gauge (ZNLBM-IIX-20 KG). A programmable electrometer (Keithley 6514) was employed to measure output voltage. Silent speech signals were acquired using an NI 1252A readout electronic on the PyCharm platform. The CNN-LSTM model was developed based on the PyTorch framework and trained on a GeForce RTX 4070 GPU. The UI interface is designed based on PyQt, and the mobile application is developed using App Inventor.

## Results and Discussion

### Design of the RT-SSRS and the FPS

**Fig. 1:**
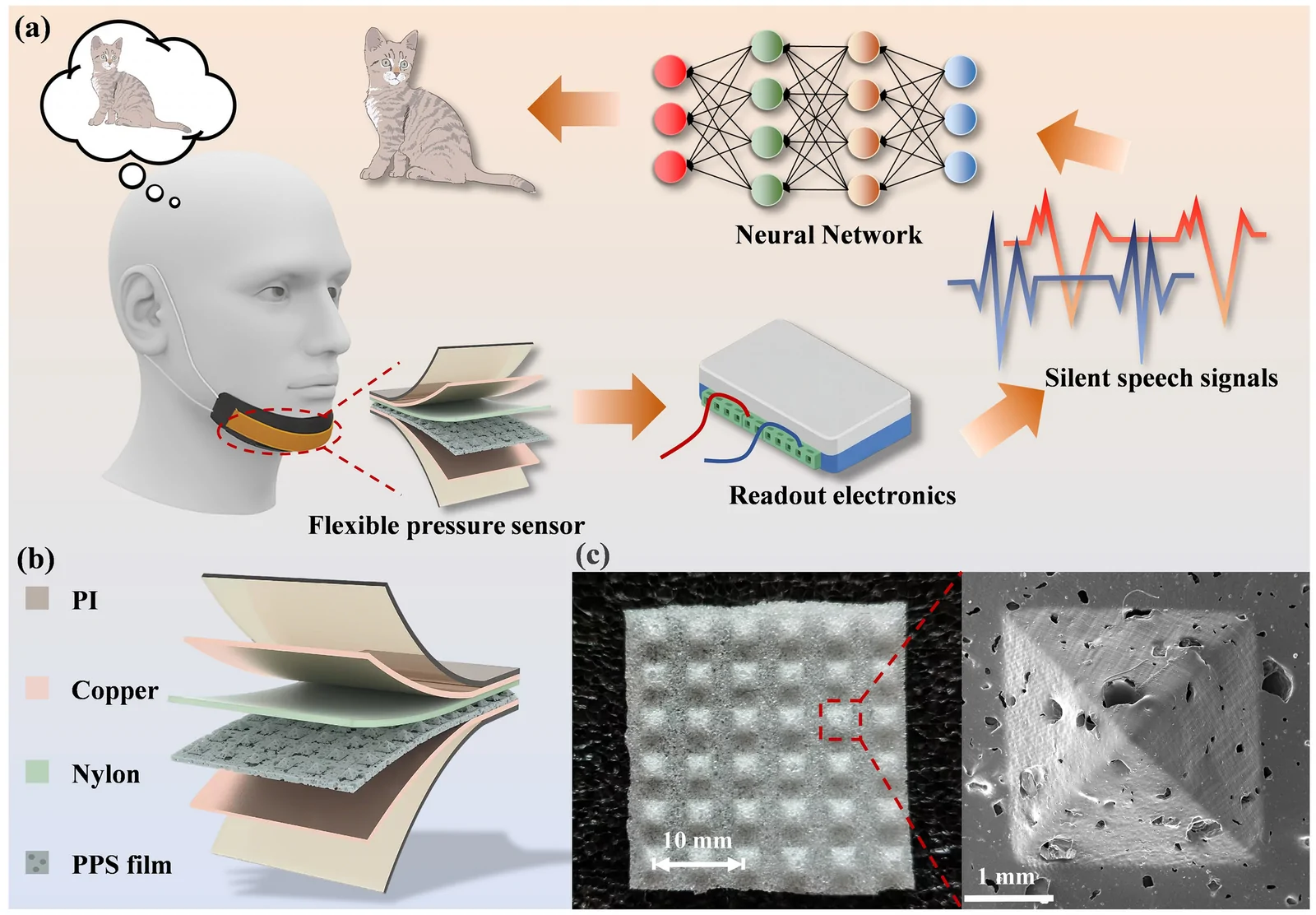
Design of the RT-SSRS and the FPS. **a** Overall architecture of the RT-SSRS. **b** Schematic representation of the structure of the FPS. **c** Top view and SEM image of the PPS film

The architecture of RT-SSRS is shown in Fig. 1a. The RT-SSRS consists of a FPS, a readout electronic, and a neural network. The FPS is worn on the chin and is capable of converting pressure variations caused by muscle movements into electrical signals. The readout electronic module is responsible for transmitting the signals. Finally, the signals are input into a trained neural network model, enabling precise and real-time decoding of silent speech signals.

Recent research advancements have highlighted the critical importance of microstructural engineering in enhancing the performance of flexible electronic devices. As systematically reviewed by Huang et al., the importance of microstructural engineering in flexible metamaterial electronics provides key design guidelines for achieving high-performance sensing [38]. Here, we fabricated FPS by combining PPS film with copper foil, nylon and polyimide (PI) in the structure shown in Fig. 1b. PPS film is used as the negative triboelectric layer, which has excellent triboelectric electrical properties, elasticity, durability and biocompatibility, and can be customized with different surface morphologies. Nylon is used as a positive triboelectric layer because it shows the highest output voltage in triboelectric electrical performance test. Copper foil is used as an electrode, and PI is used to encapsulate the entire sensor to reduce interference from sweat and human body potential. The materials used are low-cost and easy to process for mass production, and the sensor is lightweight and comfortable to wear with little extra burden.

This study employs a simple and efficient sacrificial NaCl templating method for the preparation of PPS film (Fig. S1). First, NaCl was thoroughly mixed with Ecoflex 00–30 material and poured into a pyramid-structured mold for curing. The PPS film is subsequently obtained by dissolving the NaCl particles and drying the material. The detailed steps of this preparation process are described in the experimental section. The PPS film can be stretched and twisted (Fig. S2), indicating its good mechanical properties. The PPS film is illustrated in Fig. 1c, which includes a top-view image showing a uniformly distributed pyramid structure that deforms under external forces, and an SEM image revealing a porous microstructure that helps reduce its elastic resistance. The film exhibits excellent mechanical and triboelectric properties, enabling it to detect subtle pressure variations.

The working principle of FPS adopts the contact separation mode (Fig. S3). The whole cycle is divided into four stages: first, when the muscle squeezes the FPS, the PPS film comes into contact with the nylon. Owing to the different electronegativity of the two triboelectric layers, negative triboelectric charges are generated on the PPS film side, while positive triboelectric charges are generated on the nylon side (Stage 1). When the muscle contracts, the two triboelectric layers begin to separate, and due to the potential difference generated by electrostatic induction, which drives electrons to flow in the external circuit from the PPS film electrode to the nylon electrode (stage 2). Until the muscle completely contracts, the external force disappears, and the two triboelectric layers reach the maximum separation distance (stage 3). When the muscle starts to squeeze the FPS again, the two triboelectric layers come close to each other, driving the flow of electrons from the nylon side to the PPS film side, generating a reverse current (stage 4). Once the muscle completely squeezes the FPS, this cycle returns to stage 1, completing one cycle. It can be seen that the FPS is self-powered and does not require an external power source to generate a voltage output, which is one of its main advantages over other sensors.

### Electrical Characteristics of the FPS

**Fig. 2:**
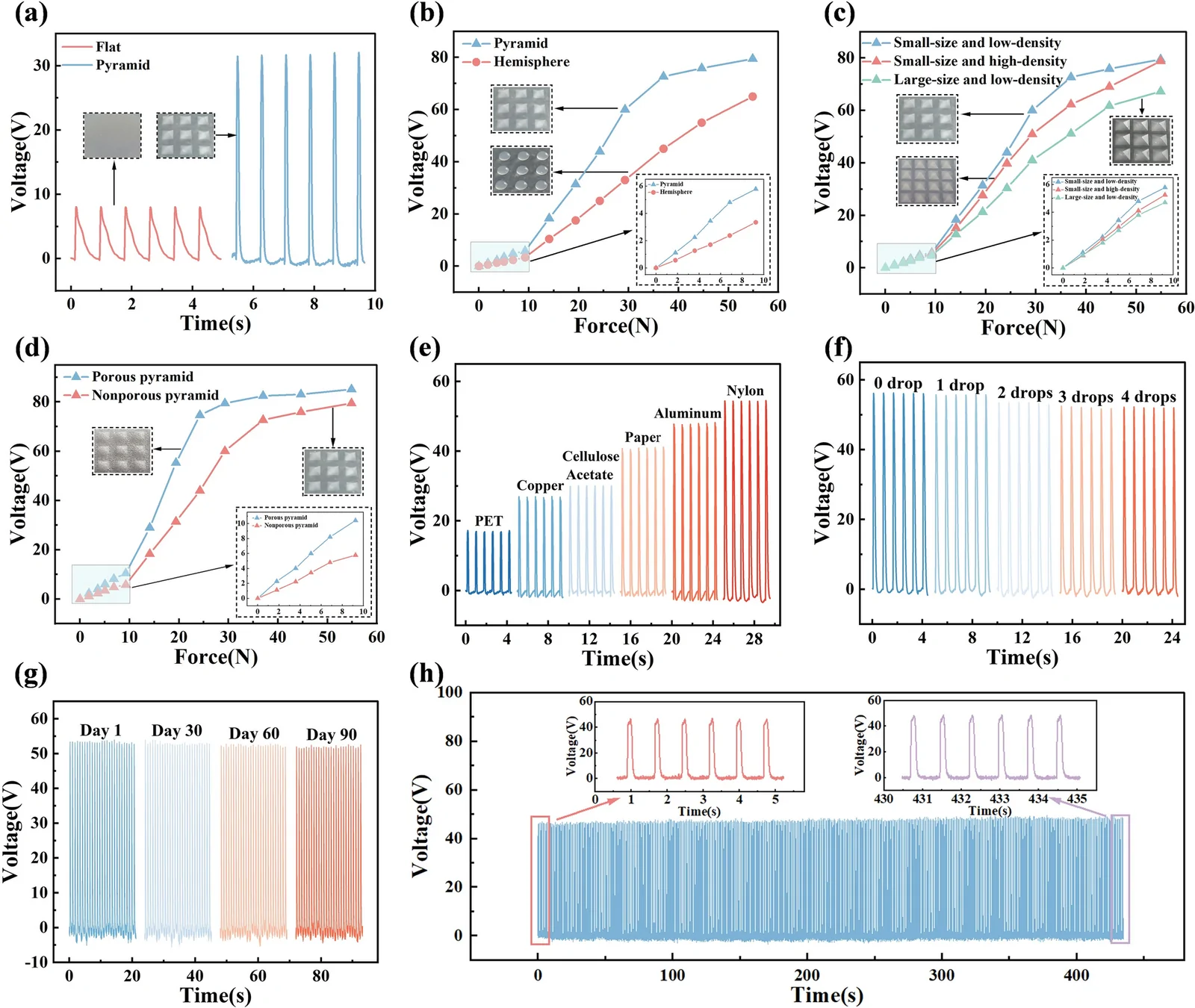
Electrical characteristics of FPS. **a** Comparison of open-circuit voltage between pyramid and flat structures under the same pressure. **b** Pressure response of pyramid and hemisphere structures. **c** Pressure response of pyramid structures with different sizes and densities. **d** Pressure response of porous pyramid structure and non-porous pyramid structure. **e** Output voltage of FPS using different positive triboelectric materials. **f** Output voltage of FPS under different amounts of artificial sweat. **g** 90 days durability test. **h** 580 cycles stability test

The surface structure of FPS has a significant impact on its pressure response characteristics. We fabricated silicone films as the negative triboelectric layer with different surface structures using various molds, with nylon as the positive triboelectric layer. First, we compared the open-circuit voltage of FPS with flat and pyramid structures under the same pressure (29 N). As shown in Fig. 2a, the output voltage of pyramid structure (31 V) is about four times that of the flat structure (8 V). This result can be attributed to the following factors: (1) This is because the capacitance change in the deformation process is significantly improved due to the presence of the air voids and the increase in effective dielectric constant [39]. (2) In the structured films, the triboelectric charges are more easily separated and thus a larger dipole moment will form between the electrodes [40].

Figure 2b shows the pressure response curves of FPSs with different surface structures (pyramid and hemisphere). The output voltage increases with pressure, as higher pressure deforms the surface, enlarging the contact area between triboelectric layers and generating more charges. The curves exhibit several nearly linear intervals. In the 0–10 N range, the pyramid structure shows higher sensitivity (0.58 V N^− 1^) than the hemisphere structure (0.33 V N^− 1^). In the 10–35 N range, both sensitivities increase, with the pyramid structure (2.8 V N^− 1^) remaining superior to the hemisphere structure (1.67 V N^− 1^). Beyond 35 N, the pyramid structure saturates due to full deformation, while the hemisphere structure continues to rise steadily owing to its higher elastic resistance.

To further investigate the effect of surface geometry, three pyramid-structured FPSs with different sizes and densities were fabricated: (1) small-size, low-density (1.5 mm height, 3 mm width, 3 × 3 array), (2) large-size, low-density (2 mm height, 4 mm width, 3 × 3 array), and (3) small-size, high-density (1.5 mm height, 3 mm width, 4 × 4 array). The small-size, low-density structure corresponds to that in Fig. 2b. As shown in Fig. 2c, the size and density of the FPS affect both its sensitivity and pressure range. A trade-off must be considered for practical applications. For silent speech recognition, we selected the small-size, low-density structure, as it offers the highest sensitivity while maintaining a suitable pressure range for this scenario.

As can be seen, adjusting the shape, size, and density of surface structures can reduce elastic resistance and increase voltage output under the same pressure. However, the accompanying reduction in overall surface area limits the growth of effective contact area, resulting in only modest improvements in pressure sensitivity. To overcome this limitation, we fabricated a porous structure based on the small-size, low-density pyramid design. This approach not only lowers elastic resistance but also increases the specific surface area [41], significantly enhancing pressure sensitivity. As shown in Fig. 2d, the porous pyramid exhibits markedly higher sensitivity than the non-porous counterpart (1 V N^− 1^ for 0–10 N and 4.6 V N^− 1^ for 10–24 N), with an overall response range of 0–24 N, making it suitable for silent speech signal acquisition.

The triboelectric properties of the positive layer directly influence the efficiency of charge generation. Figure 2e compares the output voltage of FPSs using different materials as the positive triboelectric layer. Nylon exhibits a significantly higher output voltage than other materials, due to its stronger tendency to lose electrons in the triboelectric series, generating more charges during contact–separation. Therefore, nylon was chosen as the positive triboelectric material.

In practical applications, sweat on the skin surface may affect the triboelectric properties of the FPS. Figure 2f shows the output voltage of the FPS as the amount of artificial sweat increases. The FPS exhibited a slight voltage drop, verifying its suitability for use in high-humidity environments. In addition, temperature-dependent tests (Fig. S4) reveal that the output voltage slightly increases with rising temperature but remains overall stable. Since the FPS is worn in close contact with the human body, the temperature variations are limited; within the physiological range of 23.6–42.3 °C, the output voltage remains stable, further demonstrating reliable performance under practical thermal conditions.

To further assess the long-term reliability of the FPS, a 90-day endurance test was conducted, with output voltage measured at 1, 30, 60, and 90 days. The voltage remained stable (Fig. 2g), and after 580 contact–separation cycles, the output was well maintained (Fig. 2h). SEM images of the pyramid structures after cycling (Fig. S5) show that the morphology remains intact without noticeable deformation. Considering that the FPS is mainly used to detect jaw and surrounding muscle movements in silent speech recognition, which involve relatively low and intermittent pressures, the pyramid structures are unlikely to experience significant stress, further ensuring long-term stability.

### Acquisition and Analysis of Silent Speech Signals

During actual speech, jaw movements and local muscular activities exert pressures on the FPS surface, driving the contact–separation process of the triboelectric layers and thereby inducing dynamic charge transfer. Specifically, jaw closing or local muscle contraction, corresponding to the separation process (Stages II–IV in Fig. S3), drive electrons from the PPS film electrode to the nylon electrode. If the PPS film electrode is defined as the positive terminal, this results in a negative voltage spike. In contrast, jaw opening or muscle relaxation, corresponding to the contact process (Stages IV–II in Fig. S3), induce electron transfer in the opposite direction, and generate a positive spike. Consequently, the sequence of jaw motions is directly converted into a characteristic voltage waveform. The resulting voltage waveform accurately reflects the temporal characteristics of muscle movements during speech, providing a reliable electrical signal foundation for subsequent speech pattern recognition and classification.

**Fig. 3:**
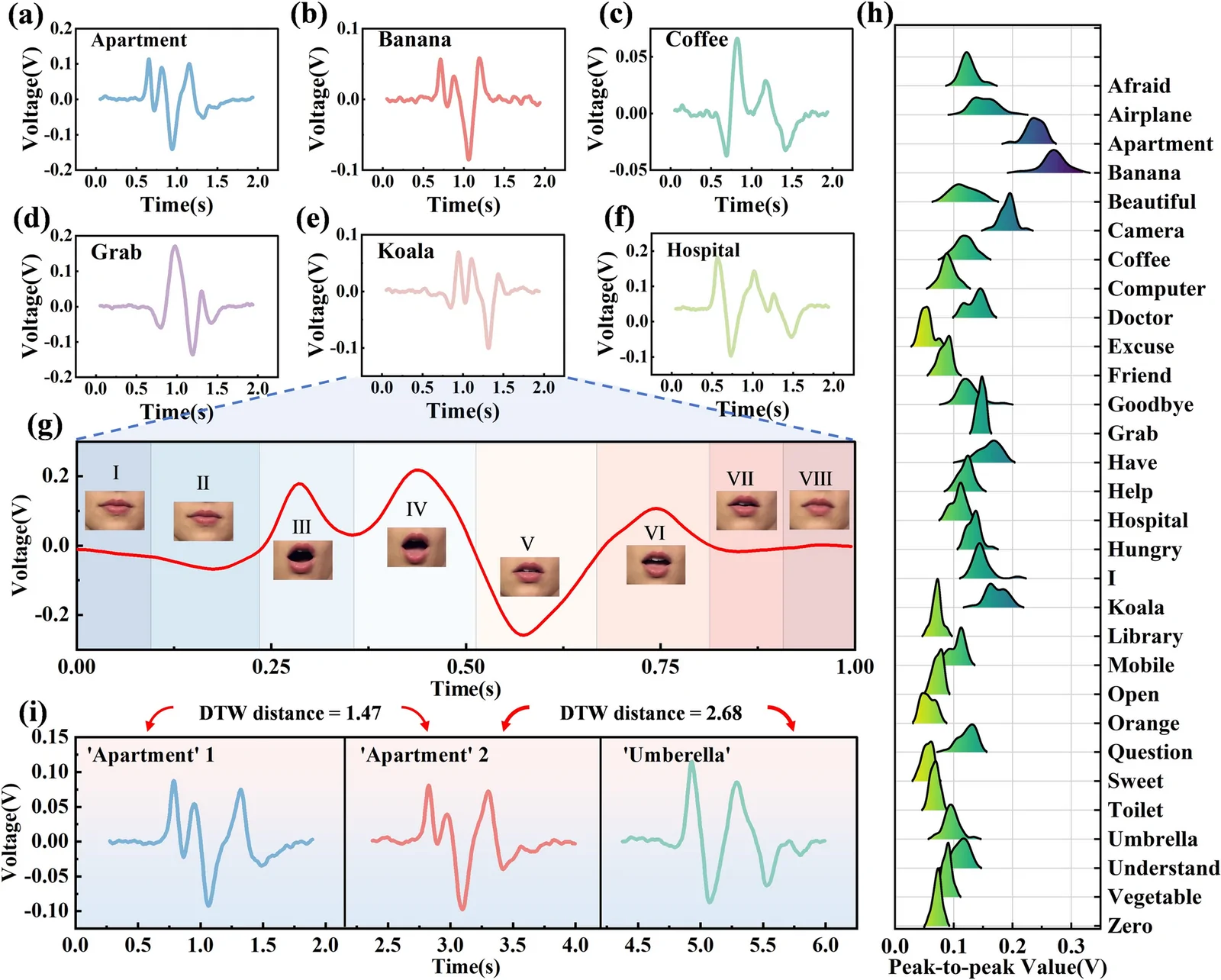
Acquisition and analysis of silent speech signals. **a-f** Preprocessed waveforms of six selected silent speech signals. **g** Correspondence between silent speech signal and mouth movements, divided into eight phases for “Koala” as an example. **h** Ridge plot of peak-to-peak feature of silent speech signals. **i** Comparison of DTW distances between intra-class (“Apartment” and “Apartment”) and inter-class (“Apartment” and “Umbrella”)

In order to investigate the characteristics of the silent speech signals captured by FPS, we analyzed 30 categories of daily words as representative samples for analysis. To ensure the validity and accuracy of the subsequent analysis, the silent speech signals were preprocessed to preserve the original signal characteristics and reduce the interference from external factors. First, the raw signals were passed through a low-pass filter with a cutoff frequency of 20 Hz to filter out work-frequency interference and other high-frequency noise. Next, the baseline was removed to eliminate slow drift due to environmental changes or physiological factors to ensure signal stability. Figure 3a–f shows the preprocessed silent speech signals for six selected categories, while signals for all 30 categories are shown in Fig. S6.

It is worth noting that to achieve real-time performance and portability, we employed a data acquisition (DAQ) card to replace electrometers for signal acquisition, which resulted in a reduction in signal amplitude (Note S1 and Fig. S7). Furthermore, the output voltage waveforms acquired initially and after 10 h of wear exhibit high consistency without noticeable deformation (Fig. S8). This demonstrates that the FPS can ensure stable signal acquisition and reliable performance during extended use.

As shown in Fig. 3g, the word “Koala” can be divided into multiple stages, with jaw contractions, openings, and closures producing characteristic positive and negative spikes. During Stage 1, the subject prepares to pronounce the syllable, and the signal remains at baseline. Stage 2, preparing /kəʊ/, shows a significant negative spike due to initial muscle contraction. Stage 3, during /kəʊ/ emission, produces prominent positive spikes from mouth opening and associated muscle activity. Stage 4, immediately after /ɑː/, exhibits a second positive spike before returning toward baseline. Stage 5, during the transition to /lə/, shows a negative spike from oral cavity closure. Stage 6, pronouncing /lə/, generates small positive spikes. Stage 7, at the end of articulation, produces small negative spikes as the mouth closes. Stage 8, after pronunciation completion, sees muscle activity cease and the signal return to baseline. This confirms that the FPS is capable of reliable mapping between articulation dynamics and electrical signals.

In order to further understand the silent speech signals, we constructed a database containing 120 samples for each class. We chose three shallow features, duration, spectral centroid and peak-to-peak value, for statistical analysis of the samples in the database. Figure 3h shows the ridge plot of the peak-to-peak value feature. It can be seen that the distribution differences between some categories are significant. For example, “Banana” is predominantly distributed around 0.26 V, while “Grab” is concentrated around 0.14 V. However, there is significant overlap in the distribution of other categories such as “Toilet” and “Zero”, which are predominantly distributed around 0.075 V. The same is true for duration and spectral centroid (Fig. S9), where the distributions of certain categories show some differentiation, but there are also many categories with varying degrees of overlap between them. Therefore, classification of silent speech signals cannot be effectively performed by simply setting thresholds for these surface features.

To further investigate the similarity of silent speech signals in the database, we employed Dynamic Time Warping (DTW) distance as a metric. DTW effectively handles nonlinear distortions and differences in sequence length, making it suitable for evaluating silent speech signal similarity. Taking "Apartment" and "Umbrella" as examples. As shown in Fig. 3i, the DTW distance between the "Apartment" samples (1.47) is smaller than that between "Apartment" and "Umbrella" samples (2.68), indicating higher similarity within the same category. We then computed average DTW distances across all categories (Fig. S10). The diagonal elements are the smallest in their respective rows and columns, confirming that intra-class similarity is higher than inter-class similarity. This demonstrates the stability of the FPS and its effectiveness in capturing distinctive silent speech features. However, some category pairs exhibit relatively low average DTW distances, with the minimum reaching 1.1, suggesting high similarity that could lead to classification errors. These findings highlight the need for more advanced classification methods to further improve silent speech recognition performance.

### Design and Performance of the Neural Network

As analyzed above, some classes of voltage signals exhibit high similarity. Therefore, ensuring clear and accurate classification of different words has been one of our primary challenges, which is why we introduce the neural network. To further improve recognition performance, we developed a hybrid neural network combining convolutional neural network (CNN) and long short-term memory (LSTM), which enables extraction of both local spatial features and temporal dynamic patterns of the signals. Instead of simply comparing waveform similarity, the model takes into account statistical indicators such as mean, variance, and power spectral density, thereby capturing deeper mappings between different wave forms and their corresponding vocabularies. Because of this approach, even if two wave forms present similar signal patterns, the statistical differences allow us to accurately determine their respective words. For more complex and highly similar vocabularies, future work may require leveraging contextual information together with large language models to address the issue.

**Fig. 4:**
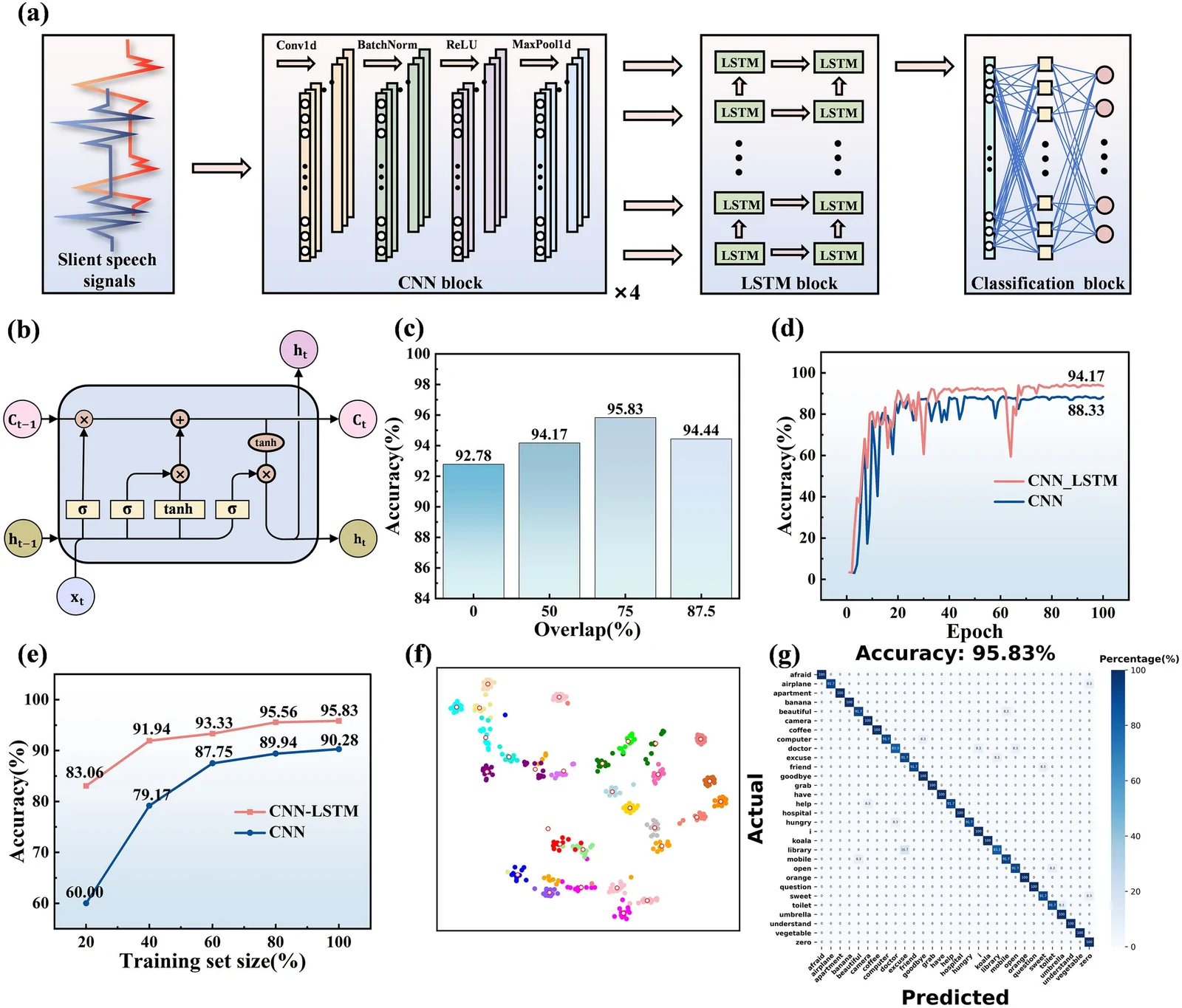
Design and performance of the CNN-LSTM neural network. **a** Schematic diagram of the structure of the CNN-LSTM. **b** The internal structure of the LSTM unit. **c** Accuracy under different sliding window step sizes. **d** Validation accuracy curves of CNN and CNN-LSTM during training. **e** Accuracy of the CNN-LTM under different training set sizes. **f** t-SNE visualization of feature embeddings from the CNN-LSTM. **g** Confusion matrix of the CNN-LSTM for 30 categories of daily words

The structure of the CNN-LSTM is shown in Fig. 4a. It mainly consists of three modules: the CNN block, the LSTM block, and the classification block. The CNN block serves as the front end, capturing local features within a short time window, such as waveform peaks, valleys, and slopes. The LSTM block, whose unit structure is shown in Fig. 4b, receives the extracted features from the CNN block and performs dynamic temporal modeling. Finally, the processed features are passed to the classification block, where a fully connected layer maps them to the output space, and a softmax activation converts the results into a probability distribution.

The dataset is crucial to the performance of the neural network, and the process of creating the dataset is as follows: Take "Koala" for example, participants were asked to say "Koala" once every 2 s, 30 times for each group, 10 groups in total. Then, the data were preprocessed (filtered and de-baselined). Finally, the overlapping sliding window is applied to segment the data.

To evaluate the effectiveness of the overlapping sliding window, we applied different step sizes to the training set and tested the resulting CNN–LSTM performance (Fig. 4c). As the overlap ratio increased, test accuracy gradually improved, peaking at 95.83% with a 75% overlap. However, further increasing the overlap to 87.5% reduced accuracy to 94.44%. This demonstrates that overlapping windows effectively increase the number of training samples and improve the robustness of the model. Nevertheless, overly small step sizes introduce redundant samples, leading to overfitting and higher computational costs. Based on this trade-off, we selected a 100-step window (75% overlap), which balances data augmentation and sample diversity while avoiding redundancy.

To validate the contribution of the LSTM module, we compared CNN and CNN-LSTM in classifying 30 daily words. As shown in Fig. 4d, both models’ validation accuracy curves converge rapidly, but CNN-LSTM achieves a higher final accuracy of 94.17% compared to 88.33% for CNN, demonstrating its superior performance. To further evaluate the models’ dependence on training set size, we gradually reduced the training samples to 100%, 80%, 60%, 40%, and 20%. While accuracy declines for both models with fewer samples, CNN-LSTM remains more stable, maintaining 83.06% accuracy at 20% of the training data, whereas CNN drops to 60.00% (Fig. 4e). The t-distributed Stochastic Neighbor Embedding (t-SNE) visualization of the high-dimensional feature space shows that CNN-LSTM achieves better intra-class compactness and inter-class separability than CNN (Figs. 4f and S11a). Finally, the confusion matrix of CNN-LSTM indicates an average accuracy of 95.83%, with 17 categories reaching 100% and 11 categories above 91.7%, whereas CNN achieves only 90.28% on average with a minimum accuracy of 66.7% (Figs. 4g and S11b). These results consistently demonstrate that incorporating LSTM substantially enhances classification of silent speech signals acquired by the FPS.

To further evaluate the generalization capability of the proposed RT-SSRS, we conducted cross-individual experiments. A supplementary dataset comprising 10 daily phrases (e.g., “nice to meet you,” “thank you,” “see you later”) was acquired from three participants. Representative signals are provided (Fig. S12). And the recognition results reveal that the system achieved an average cross-individual accuracy of 91.13% across all phrase categories, with distinct clustering of signals corresponding to different phrases (Fig. S13). These results underscore the potential of the proposed approach for deployment in real-world multi-user scenarios.

Although the RT-SSRS demonstrates strong performance in individual scenarios, its cross-individual accuracy (91.13% for 10 daily phrases across three individuals) reveals the challenge of generalization. However, this result also demonstrates its potential for multi-user applications. Future work will focus on further improving the generalization and robustness of the proposed system. On the hardware side, developing higher-sensitivity sensors will facilitate the capture of subtle biomechanical variations across different users. On the algorithmic side, advanced strategies such as transfer learning, speaker adaptive modeling, and domain generalization are expected to enhance cross-individual performance.

### Application of the RT-SSRS

**Fig. 5:**
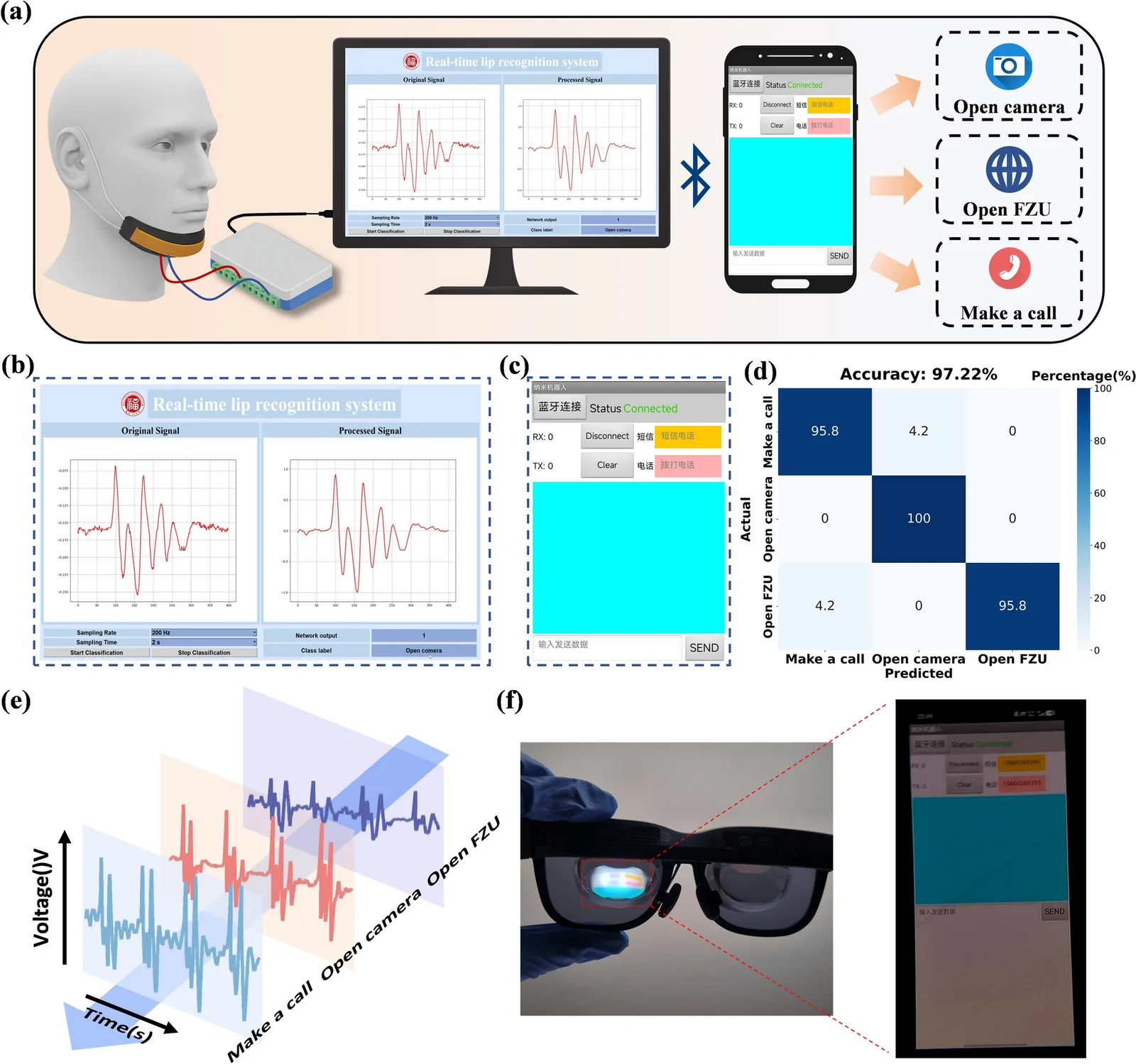
Application of the RT-SSRS. **a** Schematic illustration of RT-SSRS in a human–machine interaction scenario. **b** Computer interface displaying raw and processed signals along with recognition results. **c** Mobile application interface executing commands such as “Open camera,” “Make a call,” and “Open FZU.” **d** Confusion matrix for the three command words. **e** Waveforms of the three commands. **f** Integration with AR glasses for immersive interaction in AR/VR scenarios

To demonstrate the practical application of the RT-SSRS in human–machine interaction, we implemented a prototype system as shown in Fig. 5a. When the user utters a command, RT-SSRS recognizes and decodes the signal in real time, displays the result on the interface, and transmits the command to the smartphone via Bluetooth for execution, thereby enabling precise and contactless control. As shown in Fig. 5b, the computer interface displays both the real-time raw signals and the processed waveforms, along with the recognized command output. Figure 5c shows the mobile application interface, which performs the corresponding operation based on the received command. We implemented three representative functions: “Open camera,” “Make a call,” and “Open FZU.” The CNN-LSTM model achieved a classification accuracy of 97.22% for these commands on the test set, as illustrated by the confusion matrix in Fig. 5d. The waveform analysis in Fig. 5e demonstrates high intra-class consistency and clear inter-class distinction, confirming the system’s reliability.

A demonstration video is provided in Movie S1. As noted in Sect. 3.3, the use of a portable DAQ card resulted in a reduction of signal amplitude. Therefore, to ensure robust signal acquisition for demonstration and to better illustrate the jaw motions involved in pronouncing words, high jaw movements were employed. Furthermore, as shown in Fig. 5f, we connected the smartphone to AR glasses, enabling real-time display and interaction, which highlights the potential of RT-SSRS as a novel input modality for AR/VR applications and promotes the development of accessible and intelligent interaction technologies.

## Conclusions

In summary, this paper presents a real-time silent speech recognition system (RT-SSRS) which can acquire and decode silent speech signals in real time. The triboelectric nanogenerator (TENG)-based flexible pressure sensor (FPS), worn on the chin, detects subtle jaw movements during speech and converts them into electrical signals. Systematic characterization and optimization revealed that the outstanding performance of the FPS originates from its distinctive porous pyramid-structured silicone film (PPS) film, endowing it with high pressure sensitivity of 1 V N^− 1^ for 0–10 N and 4.6 V N^− 1^ for 10–24 N. Silent speech signals of 30 daily word categories were acquired and analyzed, revealing high similarity between classes, which demonstrates the necessity of using a neural network for accurate decoding. A hybrid deep learning framework CNN-LSTM was developed to accurately decode silent speech signals. This model achieving a classification accuracy of 95.83%, significantly outperforming the regular CNN model (90.28%). In practical human–machine interaction scenarios, the RT-SSRS enables precise and contactless control of smartphones through silent speech commands, offering a novel barrier-free communication method for individuals with speech impairments. Furthermore, by interfacing with AR glasses via smartphone, the system demonstrating strong potential for broader applications in AR/VR and related domains.

## Supplementary Information

Below is the link to the electronic supplementary material.

Supplementary file 1 (17827 KB)

Supplementary file 2 (4831 KB)
