# Supplementary material for: TENG-Based Self-Powered Silent Speech Recognition Interface: from Assistive Communication to Immersive AR/VR Interaction
Source: Nanomicro Lett. 2026 Jan 12;18:143. doi: 10.1007/s40820-025-01982-z (PMC12791100; doi:10.1007/s40820-025-01982-z)
Supplement: Supplementary file 1 — (17827 KB) [file 40820_2025_1982_MOESM1_ESM.doc]

Supporting Information for

**TENG-Based Self-Powered Silent Speech Recognition Interface: from Assistive Communication to Immersive AR/VR Interaction**

Shuai Lin^1,#^, Yanmin Guo^1,#^, Xiangyao Zeng^1^, Xiongtu Zhou^1,2^, Yongai Zhang^1,2^, Chengda Li^3^, *, Chaoxing Wu^1, 2^, *

^1^ School of Physics and Information Engineering, Fuzhou University, 350108 Fuzhou, P. R. China

^2^ Fujian Science & Technology Innovation Laboratory for Optoelectronic Information of China, 350116 Fuzhou, P. R. China

^3^ School of Artificial Intelligence, Xiamen City University, 361008 Xiamen, P. R. China

*^^*Shuai Lin and Yanmin Guo have contributed equally to this work.

*Corresponding authors. E-mail: [chaoxing_wu@fzu.edu.cn](mailto:chaoxing_wu@fzu.edu.cn) (Chaoxing Wu); [lichengda@xmcu.edu.cn](mailto:lichengda@xmcu.edu.cn) (Chengda Li)

**Supplementary Table S1** Summary of characteristics in silent speech recognition technologies

| TENG  (our work) | Radar | EMG | Vision | **Method** |
| --- | --- | --- | --- | --- |
| High (sensitive to subtle pressure variations) | Medium (may miss subtle movements) | High (direct detection of muscle activity) | High (captures most movements accurately) | **Sensitivity to Subtle Motion** |
| Lightweight wearable (integrated on chin or mask), comfortable | Contact-free (radar device) | Wearable (electrodes attached to skin), uncomfortable | Contact-free (camera) | **Wearing Method and comfort** |
| 95.83% (30 words, 1 subject), 91.13% (10 phrases, 3 subjects), self-collected dataset | 88.95% (25 words, 4 subjects), self-collected dataset | 95.63% (8 vowels), self-collected dataset | 91.8% (LRW dataset,500words), public dataset | **Accuracy** |
| Self-powered | External | External | External | **Power Supply** |
| Excellent (robust to lighting, minimally affected by temperature/humidity) | Medium (robust to lighting, affected by electromagnetic interference) | Medium (robust to lighting, affected by sweat and crosstalk) | Poor (sensitive to lighting and occlusion) | **Environment Robustness** |
|  | [S11-S15] | [S6-S10] | [S1-S5] | **Reference** |

**Supplementary Figures**


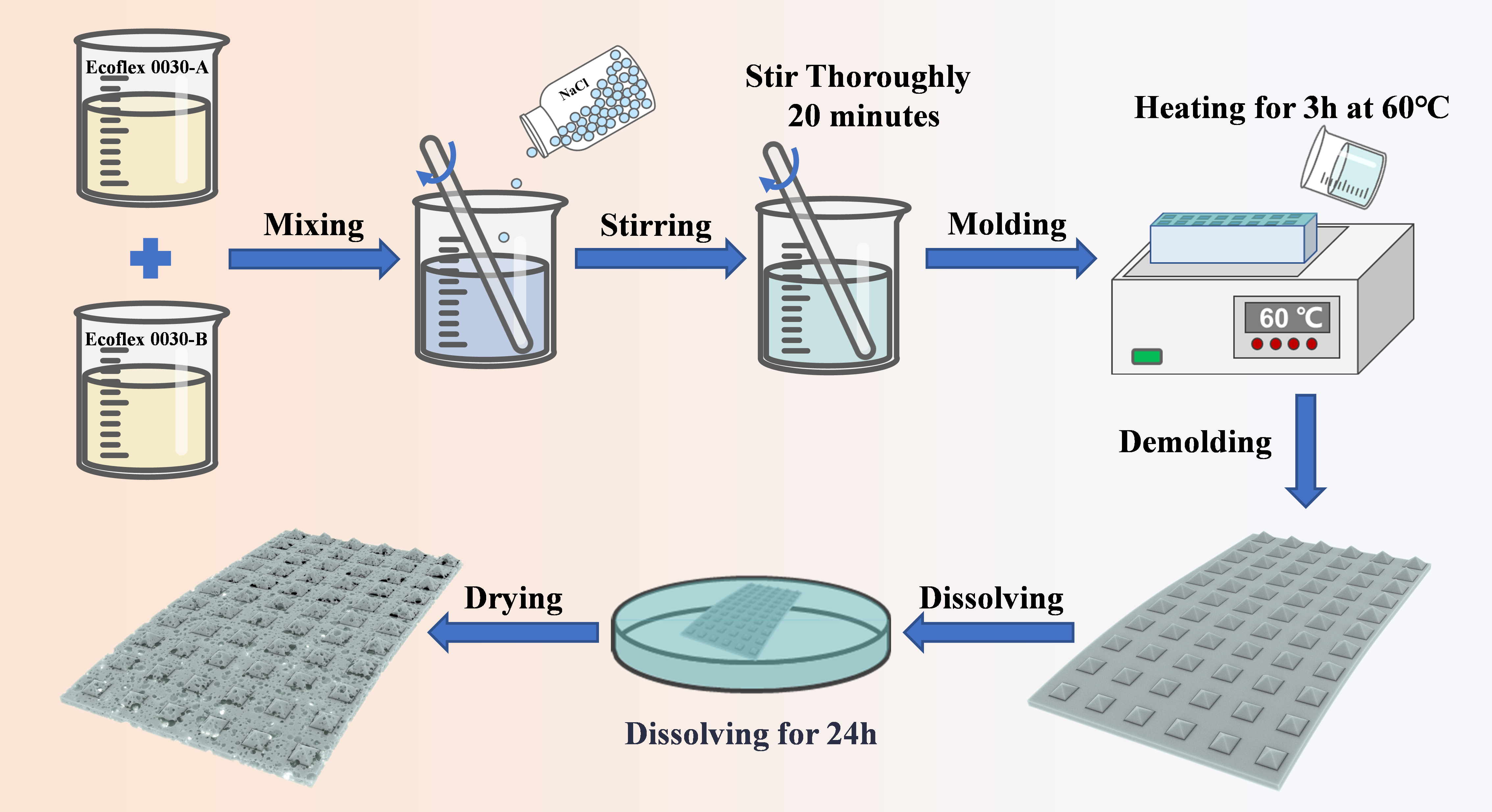


**Fig. 1** Preparation process of PPS film using sacrificial NaCl templating method.


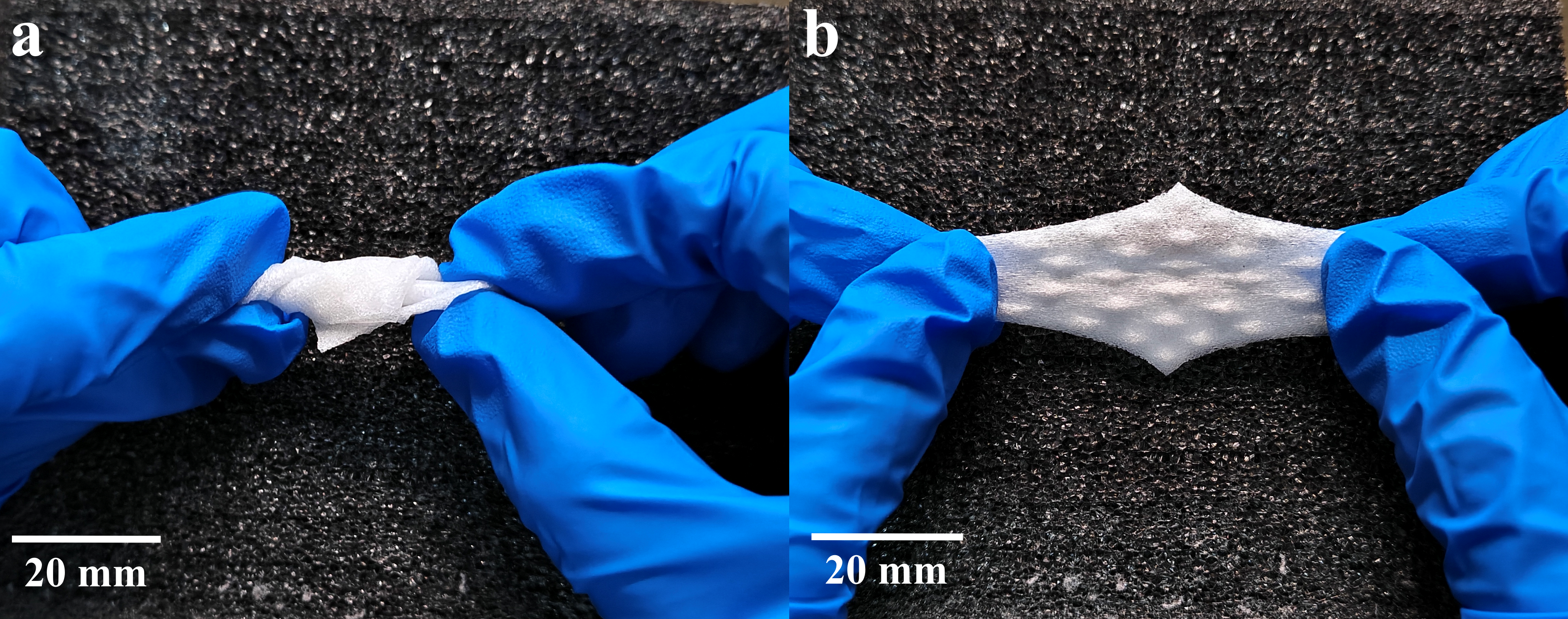


**Fig. S2** Optical images of the PPS film under twisting and stretching. a) Twisting. b) Stretching.


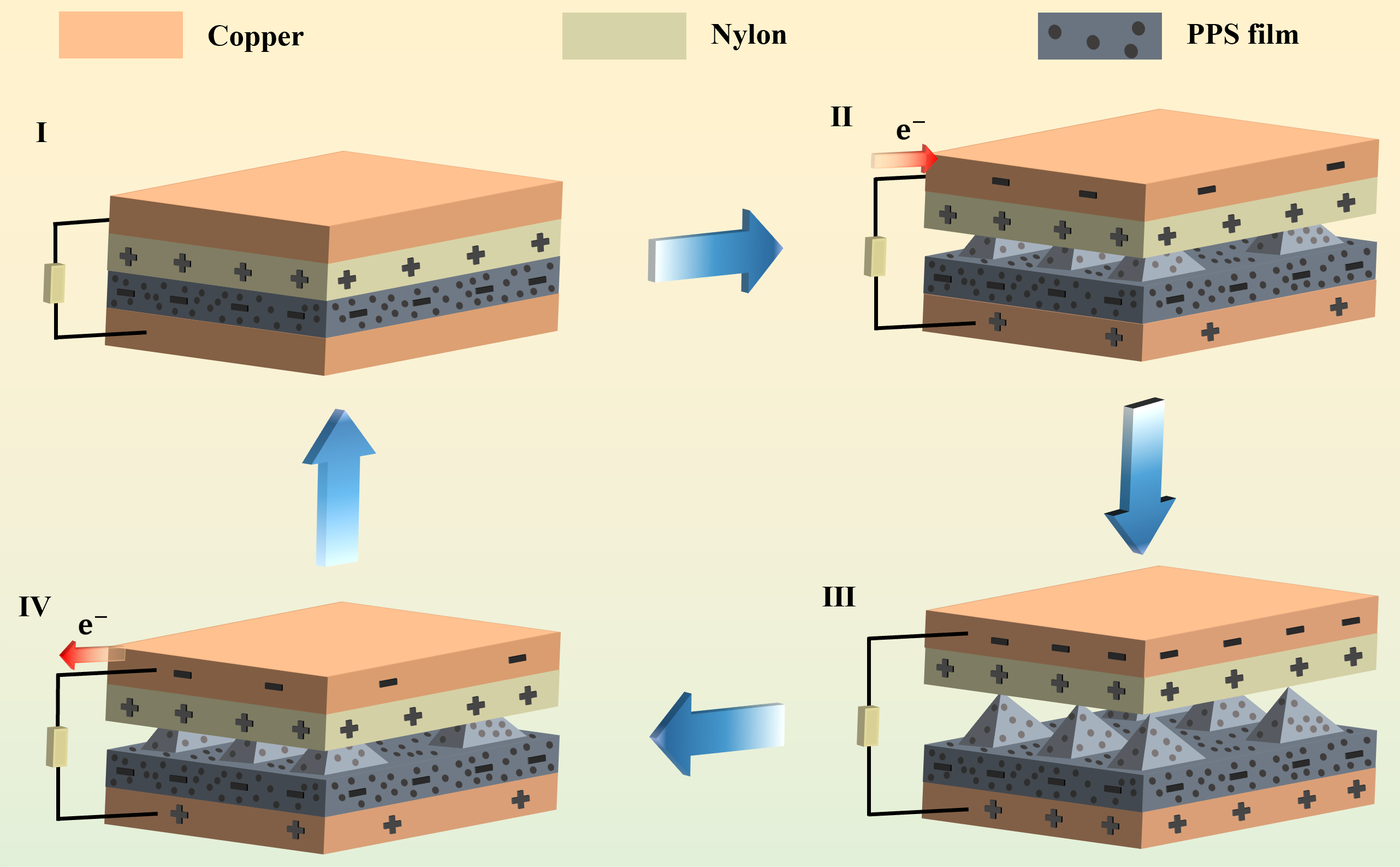


**Fig. S3** The working principle of the FPS, demonstrating the four stages of the contact separation process


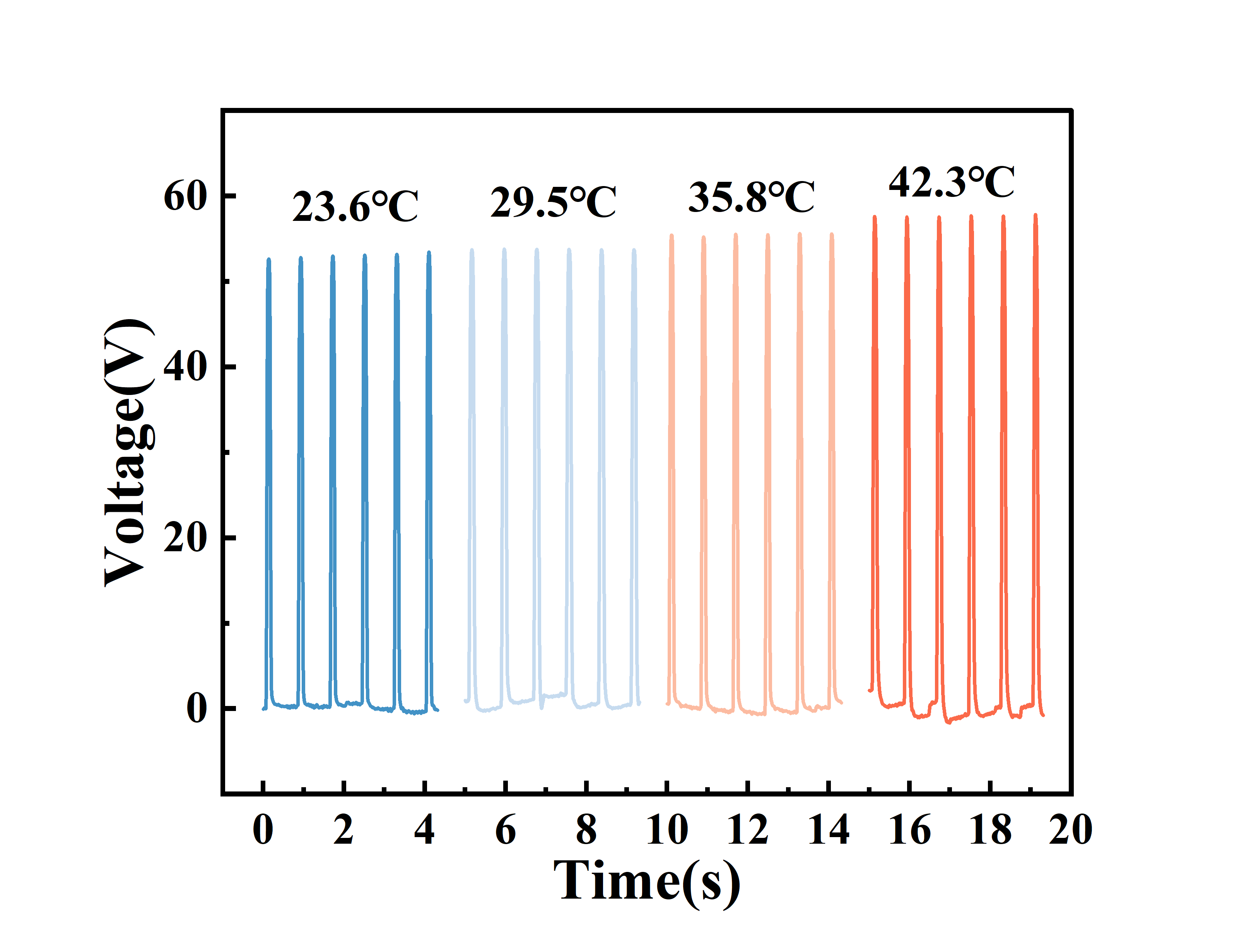


**Fig. S4** Output voltage of the FPS under different temperature


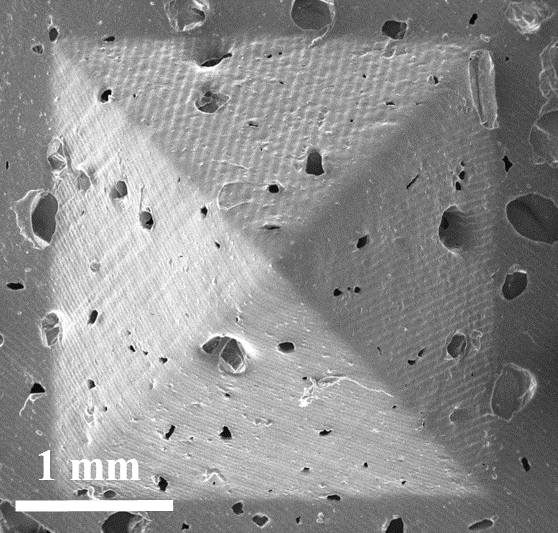


**Fig. S5** Microscopic porous pyramid structure of the PPS film under SEM after 580 contact–separation cycles


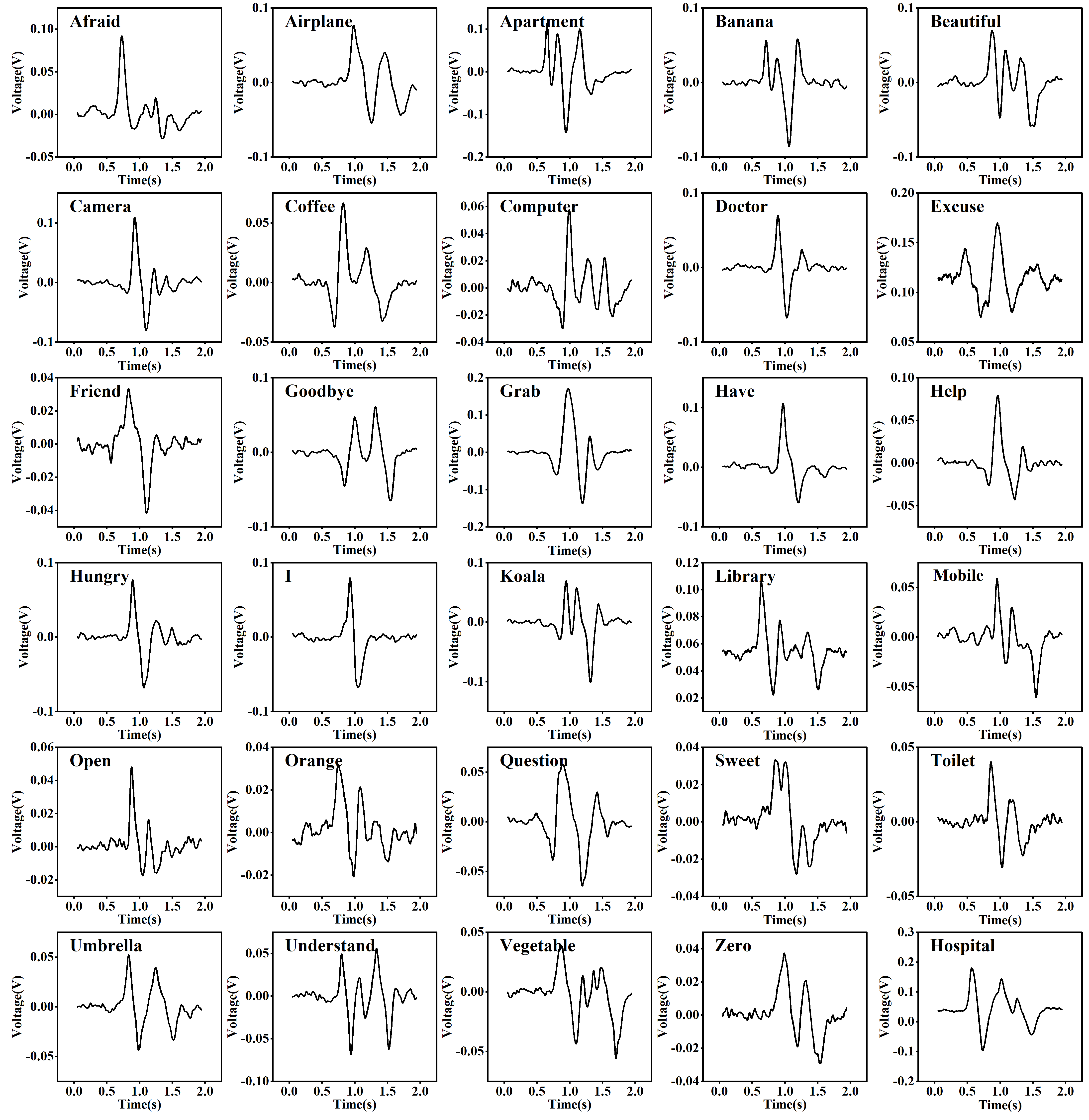


**Fig. S6** Preprocessed waveforms of 30 categories of silent speech signals

**Note S1 voltage signal acquisition**

In conventional triboelectric measurements, high-input-impedance electrometers (Keithley 6514) are typically used to capture the full potential output of the device. To enhance system real-time performance and portability, this study employs a data acquisition card (NI 1252A) for signal recording, resulting in relatively reduced voltage amplitudes. Fig. S7 shows the output voltage of the FPS recorded by DAQ card at different mouth opening amplitudes, indicating it can detect subtle jaw movements of approximately 3-4 mm (generating about 0.05 volts). This confirms the system's applicability to micro-scale phonation dynamics. Future work will focus on enhancing FPS sensitivity to reduce reliance on data acquisition electronics and optimizing the acquisition hardware to further improve user experience.


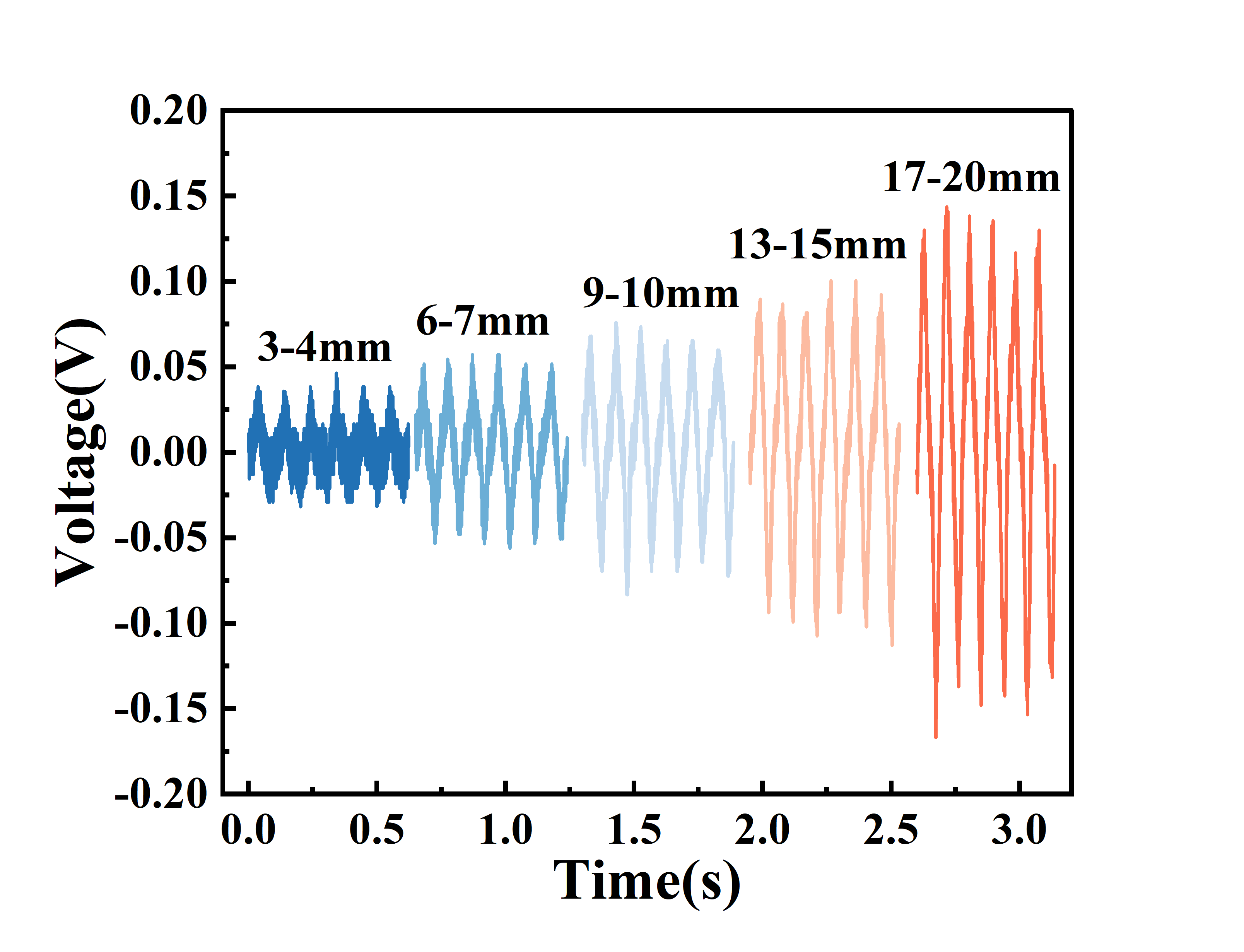


**Fig. S7** Output voltage of the FPS recorded by DAQ card at different mouth opening amplitudes


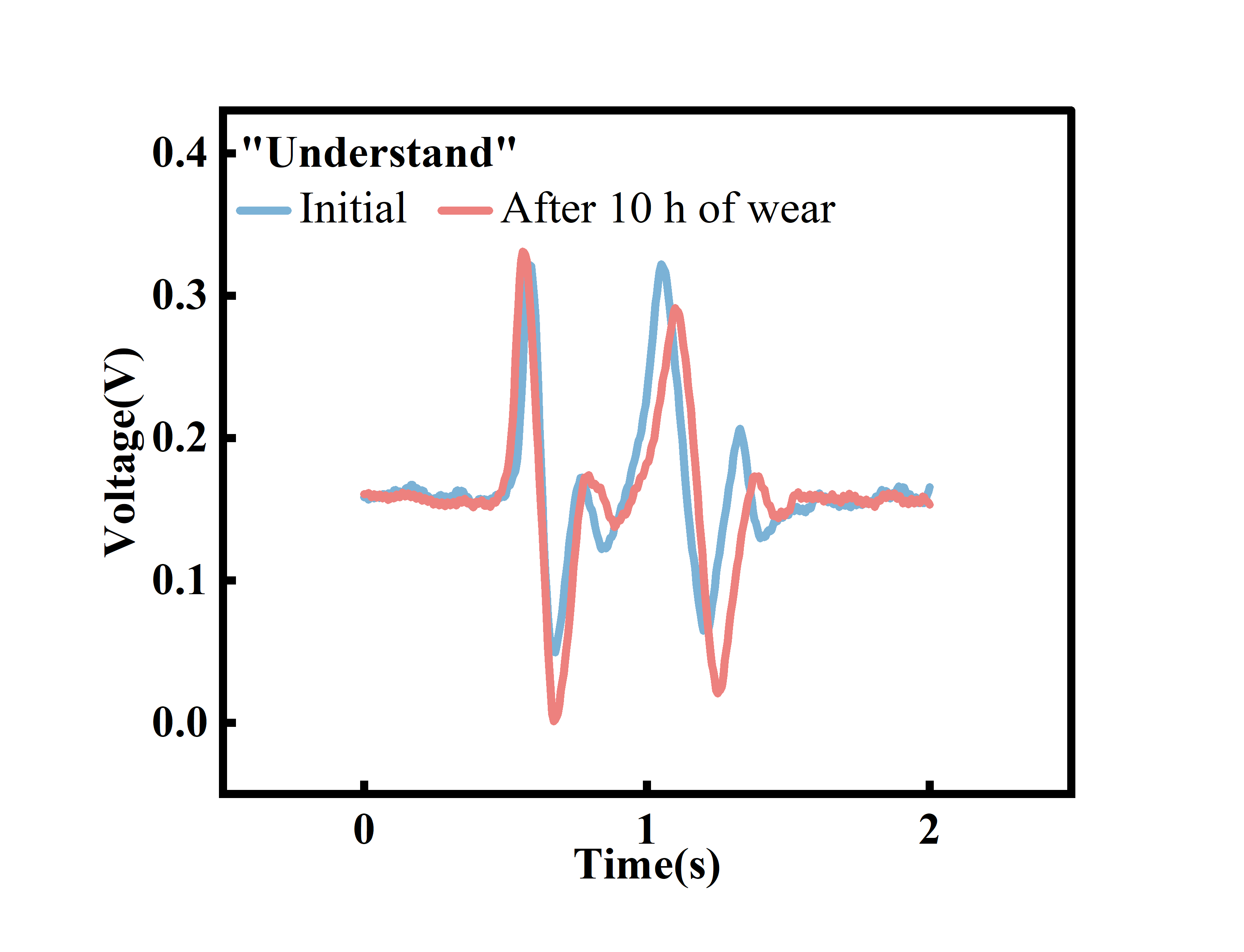


**Fig. S8** Output voltage at initialization and after 10 hours of wear


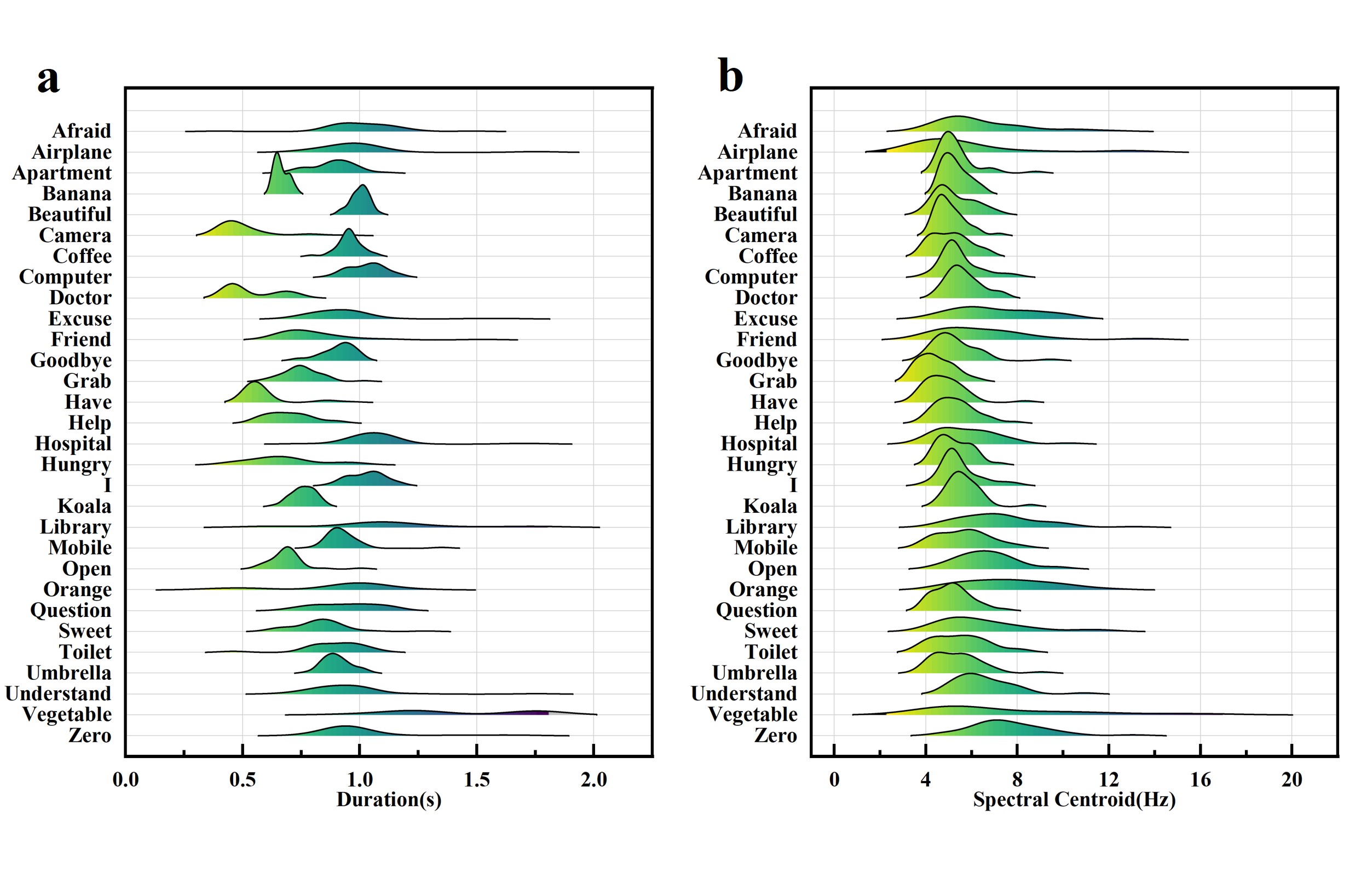


**Fig. S9** Ridge plots of duration and spectral centroid features of 30 silent speech signals. **a**) Duration. **b**) Spectral Centroid


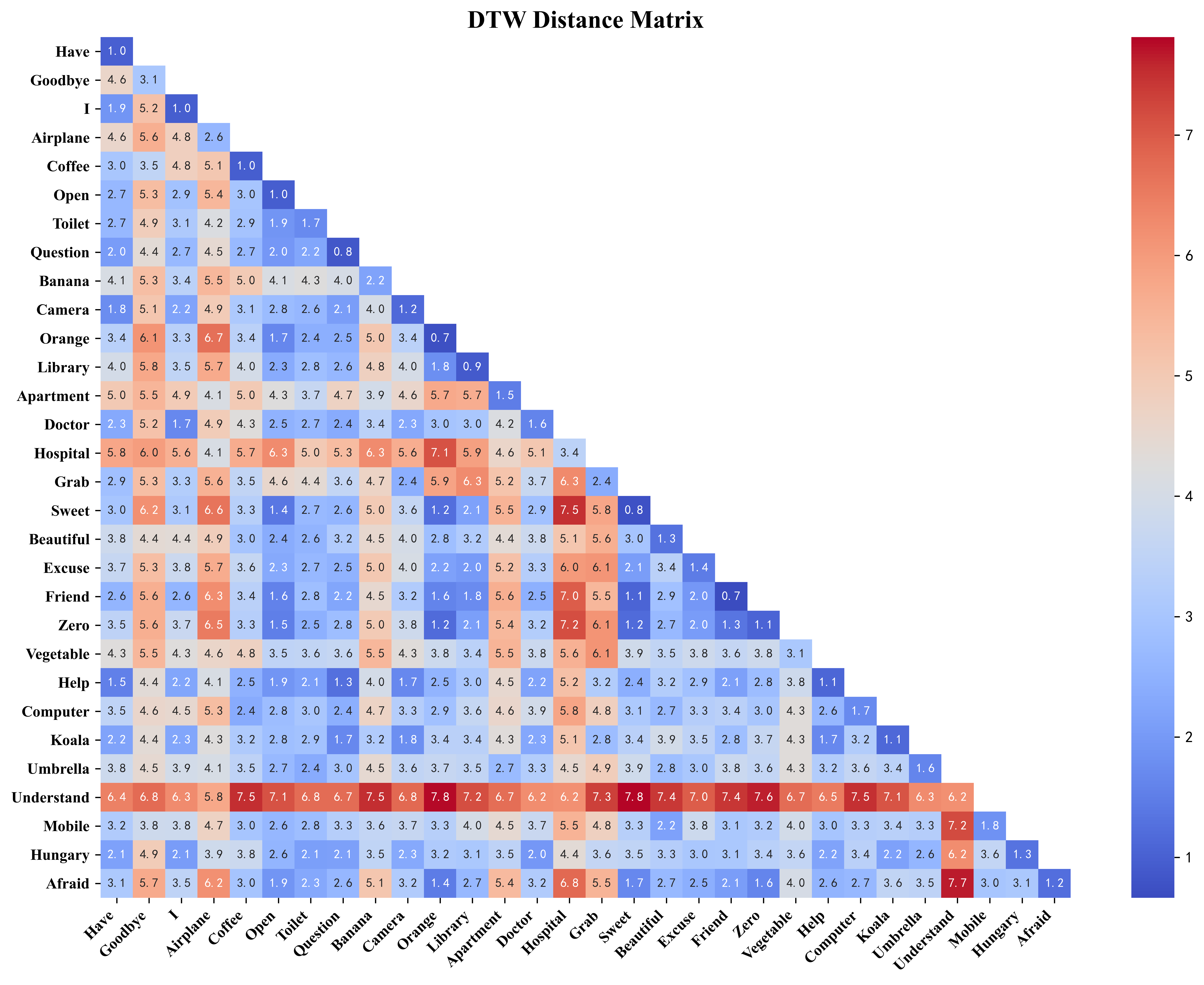


**Fig. S10** Average DTW distance matrix of 30 silent speech signals


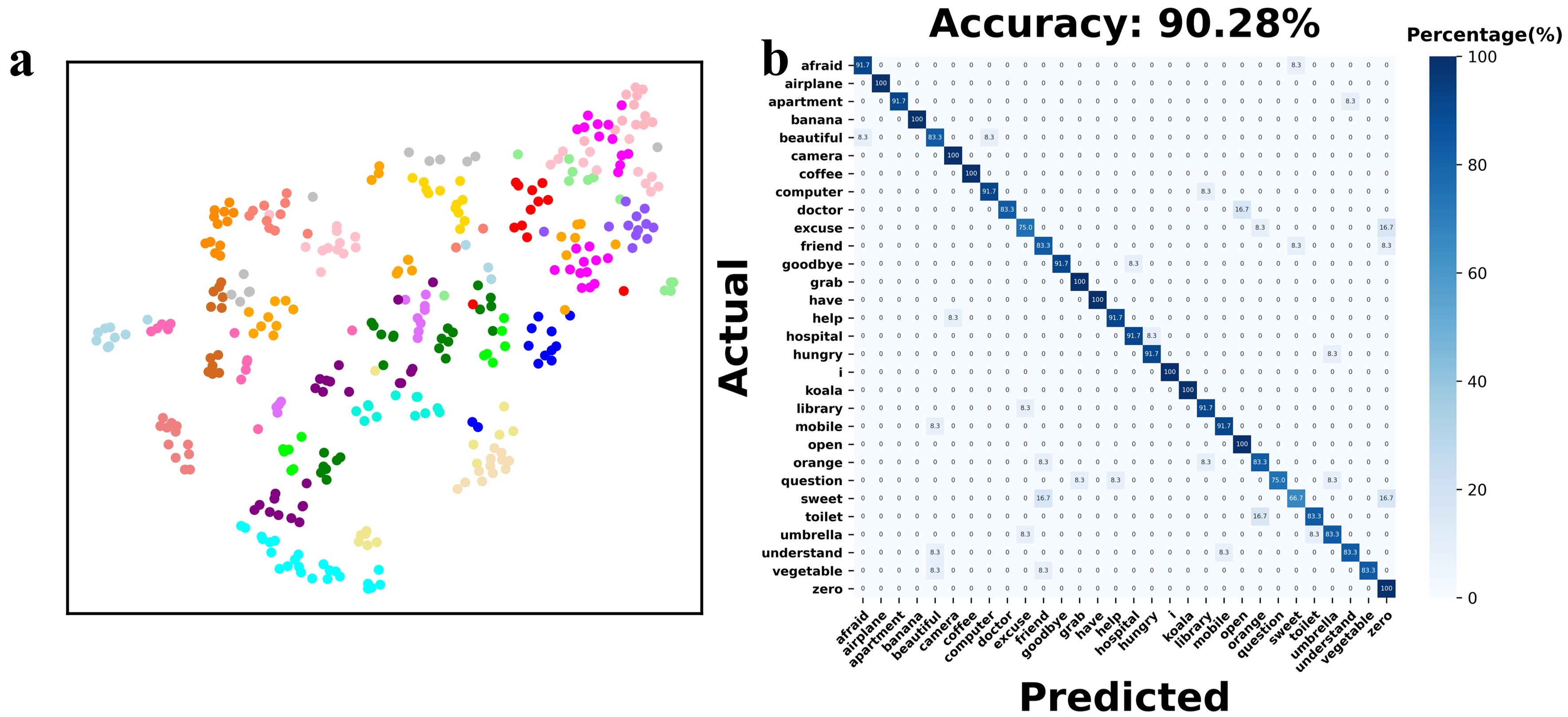


**Fig. S11 a**) t-SNE visualization distribution of samples in CNN. **b**) Confusion matrix of CNN for 30 silent speech signals


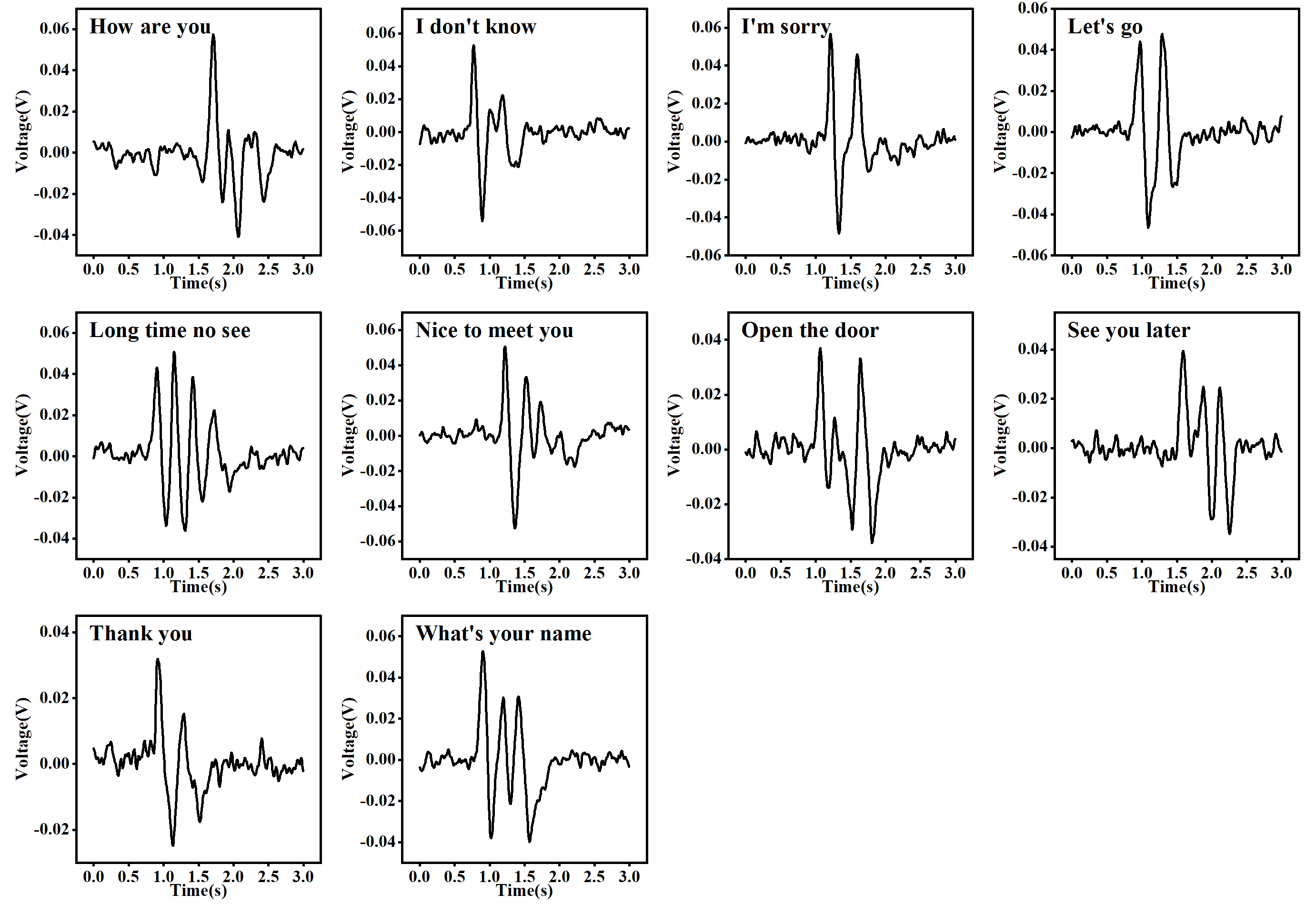


**Fig. S12** Preprocessed waveforms of 10 daily phrases signal


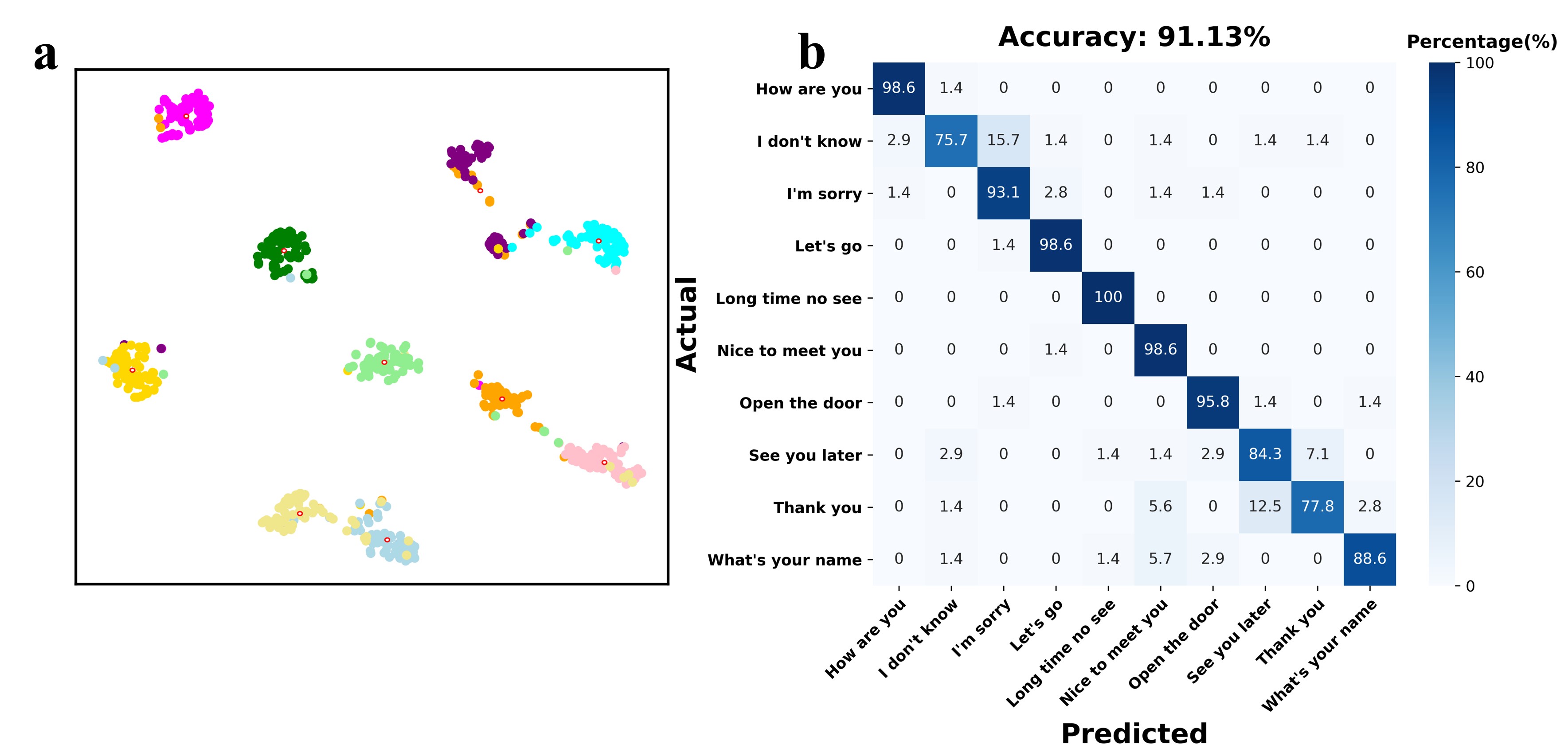


**Fig. S13** t-SNE visualization distribution and Confusion matrix of CNN-LSTM for 10 daily phrases. **a**) t-SNE visualization distribution. **b**) Confusion matrix

**Supplementary References**

1. J. Hou, Y. Zhang, X. Lu, E. Cai, K. Wei et al., An anti-occlusion vision-based method for structural motion estimation. Mech. Syst. Signal Process. **224**, 112003 (2025). <https://doi.org/10.1016/j.ymssp.2024.112003>
2. G.V. Kale, V.H. Patil, A study of vision based human motion recognition and analysis. Int. J. Ambient Comput. Intell. **7**(2), 75–92 (2016). <https://doi.org/10.4018/ijaci.2016070104>
3. C. Xia, C.-Y. Weng, Y. Zhang, I.-M. Chen, Vision-based measurement and prediction of object trajectory for robotic manipulation in dynamic and uncertain scenarios. IEEE Trans. Instrum. Meas. **69**(11), 8939–8952 (2020). <https://doi.org/10.1109/TIM.2020.2994602>
4. S.L. Colyer, M. Evans, D.P. Cosker, A.I.T. Salo, A review of the evolution of vision-based motion analysis and the integration of advanced computer vision methods towards developing a markerless system. Sports Med. Open **4**(1), 24 (2018). <https://doi.org/10.1186/s40798-018-0139-y>
5. X. Zhang, Y. Hu, X. Liu, Y. Gu, T. Li et al., A novel approach for visual speech recognition using the partition-time masking and swin transformer 3D convolutional model. Sensors **25**(8), 2366 (2025). <https://doi.org/10.3390/s25082366>
6. L. Cheng, J. Li, A. Guo, J. Zhang, Recent advances in flexible noninvasive electrodes for surface electromyography acquisition. NPJ Flex. Electron. **7**, 39 (2023). <https://doi.org/10.1038/s41528-023-00273-0>
7. F. Chamberland, É. Buteau, S. Tam, E. Campbell, A. Mortazavi et al., Novel wearable HD-EMG sensor with shift-robust gesture recognition using deep learning. IEEE Trans. Biomed. Circuits Syst. **17**(5), 968–984 (2023). <https://doi.org/10.1109/TBCAS.2023.3314053>
8. M. Rohr, J. Haidamous, N. Schäfer, S. Schaumann, B. Latsch et al., On the benefit of FMG and EMG sensor fusion for gesture recognition using cross-subject validation. IEEE Trans. Neural Syst. Rehabil. Eng. **33**, 935–944 (2025). <https://doi.org/10.1109/TNSRE.2025.3543649>
9. J.-H. Sul, L. Piyathilaka, D. Moratuwage, S. Dunu Arachchige, A. Jayawardena et al., Electromyography signal acquisition, filtering, and data analysis for exoskeleton development. Sensors **25**(13), 4004 (2025). <https://doi.org/10.3390/s25134004>
10. J.S. Kang, K.S. Moon, S.Q. Lee, N. Satterlee, X. Zuo, A wearable silent text input system using EMG and piezoelectric sensors. Sensors **25**(8), 2624 (2025). <https://doi.org/10.3390/s25082624>
11. M.T. Buyukakkaslar, M. Ali Erturk, M. Ali Aydin, A review on radar-based human detection techniques. Sensors **24**(17), 5709 (2024). <https://doi.org/10.3390/s24175709>
12. T. Roegers, Radar interference mitigation: A review. Authorea Preprints (2024).
    <https://doi.org/10.36227/techrxiv.173609886.67199763/v1>
13. C. Jin, X. Meng, X. Li, J. Wang, M. Pan et al., Rodar: robust gesture recognition based on mmWave radar under human activity interference. IEEE Trans. Mob. Comput. **23**(12), 11735–11749 (2024). <https://doi.org/10.1109/TMC.2024.3402356>
14. L. Wang, Z. Cui, Y. Pi, C. Cao, Z. Cao, Low personality-sensitive feature learning for radar-based gesture recognition. Neurocomputing **493**, 373–384 (2022). <https://doi.org/10.1016/j.neucom.2022.04.035>
15. S. Lee, Y. Shin, M. Kim, J. Seo, IR-UWB radar-based contactless silent speech recognition of vowels, consonants, words, and phrases. IEEE Access **11**, 144844–144859 (2023). <https://doi.org/10.1109/ACCESS.2023.3344177>
